# Inflammatory proteins are associated with mortality in a middle‐aged diverse cohort

**DOI:** 10.1002/ctm2.1412

**Published:** 2023-09-24

**Authors:** Nicole Noren Hooten, Nicolle A. Mode, Samuel Allotey, Ngozi Ezike, Alan B. Zonderman, Michele K. Evans

**Affiliations:** ^1^ Laboratory of Epidemiology and Population Science National Institute on Aging National Institutes of Health Baltimore Maryland USA; ^2^ Feinberg School of Medicine Northwestern University Chicago Illinois USA

**Keywords:** inflammation, midlife, mortality, race

## Abstract

**Background:**

Recent data indicate a decline in overall longevity in the United States. Even prior to the COVID‐19 pandemic, an increase in midlife mortality rates had been reported. Life expectancy disparities have persisted in the United States for racial and ethnic groups and for individuals living at low socioeconomic status. These continued trends in mortality indicate the importance of examining biomarkers of mortality at midlife in at‐risk populations. Circulating levels of cytokines and inflammatory markers reflect systemic chronic inflammation, which is a well‐known driver of many age‐related diseases.

**Methods:**

In this study, we examined the relationship of nine different inflammatory proteins with mortality in a middle‐aged socioeconomically diverse cohort of African–American and White men and women (n = 1122; mean age = 47.8 years).

**Results:**

We found significant differences in inflammatory‐related protein serum levels between African–American and White middle‐aged adults. E‐selectin and fibrinogen were significantly higher in African–American adults. IFN‐γ, TNF‐α trimer, monocyte chemoattractant protein‐1 (MCP‐1), soluble receptor for advanced glycation end‐products (sRAGE) and P‐selectin were significantly higher in White participants compared to African–American participants. Higher levels of E‐selectin, MCP‐1 and P‐selectin were associated with a higher mortality risk. Furthermore, there was a significant interaction between sex and IL‐6 with mortality. IL‐6 levels were associated with an increased risk of mortality, an association that was significantly greater in women than men. In addition, White participants with high levels of sRAGE had significantly higher survival probability than White participants with low levels of sRAGE, while African–American participants had similar survival probabilities across sRAGE levels.

**Conclusions:**

These results suggest that circulating inflammatory markers can be utilized as indicators of midlife mortality risk in a socioeconomically diverse cohort of African–American and White individuals.

## Graphical abstract

Circulating inflammatory proteins are associated with mortality in African‐American and White middle‐aged adults.

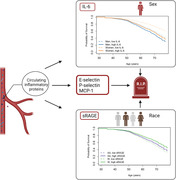

## 1 INTRODUCTION

Troubling declines in life expectancy have been recently reported for the United States (US).^1^ In the US, life expectancy dropped from 77.0 years in 2020 to 76.4 years in 2021, a loss of 0.6 years.^1^ This trend is multifactorial in nature, reflecting the excess deaths due to the COVID‐19 pandemic, but also due to other age‐related chronic diseases.^1^, ^2^ There was an increase in rates of death among children (0–19 years) and adults at midlife.^2^ This increase in mortality among US adults at midlife was reported even before the COVID‐19 pandemic and across all race and ethnicity groups.^3^, ^4^ This alarming trend indicates the importance of identifying biomarkers that may pinpoint individuals at risk for early mortality.

Recent data also report the persistent racial and ethnic disparities in life expectancy in the US.^2^ Specifically, non‐Hispanic American Indian or Alaska Native and Black populations have the lowest life expectancy in the US.^2^ Although these recent data are indicative of the harsh disparate effects of the pandemic on these populations, there have been stark disparities in Black–White mortality rates over the last century.^5^ Across high‐income countries, low socioeconomic status also remains a significant risk factor for earlier mortality.^6^ In particular, African–American men living below the poverty line are particularly vulnerable to early mortality.^5^, ^7^ In addition, the long‐standing phenomenon continues whereby women live longer than men.^1^

Chronic inflammatory diseases are a major contributor to the global burden of mortality.^8^ It is estimated that over half of the worldwide deaths can be linked to inflammatory‐related diseases^8^, ^9^ It is postulated that dampening chronic inflammatory mechanisms early and at midlife consequently leads to a reduction in morbidity and mortality.^10^ Systemic chronic inflammation is a well‐known driver of many age‐related diseases and increases susceptibility to non‐communicable diseases (rev in [^9^]). For example, in the Glasgow Inflammation Outcome Study, systemic chronic inflammation, measured using high‐sensitivity C‐reactive protein, neutrophil count and albumin, was associated with all‐cause mortality.^11^ Many circulating inflammatory markers increase with age referred to as ‘inflamm‐aging’.^12^ Advancing age results in the accumulation of senescent cells, which release many factors into the extracellular environment known as the senescence‐associated secretory phenotype (SASP).^13^ These factors drive chronic inflammation and play important roles in promoting aging and age‐related diseases and include interleukins (e.g., IL‐6), chemokines (MCP‐1) and other inflammatory molecules (IFN γ), soluble receptors (TNF‐α), non‐protein soluble protein factors and insoluble factors.^13^, ^14^

Specific inflammatory markers, most notably IL‐6, increase with age and are associated with mortality in older populations.^15^, ^16^ Few studies have examined IL‐6 levels with age and mortality in minority populations, and data have been inconsistent.^17^ Minority and low‐socioeconomic status individuals are exposed to adverse and stressful conditions that can manifest into higher levels of inflammation, leading to disparate health outcomes.^18^, ^19^, ^20^ Therefore, one aim of our study was to address the role of IL‐6 in a diverse, middle‐aged cohort to enhance our understanding of the role of inflammation in driving age‐related disease and mortality. Furthermore, examining additional inflammatory markers will help tease apart whether other inflammatory pathways are associated with mortality in minority populations. Inflammatory‐related proteins were chosen based on previous epidemiological or biological evidence that these markers may be altered by social determinants of health, age, or mortality. In particular, we chose several known SASP factors including IFN‐γ, IL‐6, TNF‐α trimer and MCP‐1.^13^, ^21^ MCP‐1 levels have also been associated with biological age in mice, and in a large meta‐analysis, higher MCP‐1 was associated with long‐term cardiovascular mortality.^22^, ^23^ E‐selectin and P‐selectin have been associated with mortality in patients with cardiovascular disease.^24^, ^25^, ^26^ Soluble receptor for advanced glycation end‐products (sRAGE) has been associated with age‐associated diseases and mortality.^27^ Higher serum amyloid A (SAA) was associated with all‐cause and cardiovascular mortality.^28^ Last, fibrinogen is a marker of thrombosis and inflammation, and has been associated with overall all‐cause mortality and in cardiovascular risk‐related mortality.^29^, ^30^, ^31^

In this prospective cohort study, we examined whether these nine different inflammatory‐related proteins were associated with mortality over an approximately 12‐year time period in a middle‐aged cohort of African–American and White men and women. As race, sex and poverty all remain risk factors for mortality, this study also explores interactions between these inflammatory proteins and these variables.

## 2 METHODS AND MATERIALS

### 2.1 Study sample

Participants were selected from the Healthy Neighborhoods of Diversity Across the Life Span (HANDLS) study of the National Institute on Aging Intramural Research Program, National Institutes of Health. HANDLS is an ongoing, prospective study that examines how race and socioeconomic status influence the development of age‐associated health disparities. The study consists of community‐dwelling non‐Hispanic African–American and non‐Hispanic White adults aged 30−64 at baseline living in Baltimore, Maryland, USA.^32^ The baseline accrual occurred between 2004−2009. Race was self‐identified as African– American or White. Participants had either household income above or below poverty as defined by 125% of the 2004 US Health and Human Services Poverty Guidelines at enrollment. Participants provided written, informed consent. The HANDLS study is approved by the Institutional Review Board of the National Institutes of Health.

For this study, 1122 African–American and White adults seen at wave 1 (2004–2009) were included who had an available fasting serum sample and had undergone a physical examination and structured medical history interview at that wave. Blood samples were collected in the morning after overnight fasting into BD Vacutainer® serum separator tubes. Samples were centrifuged at room temperature at 1142 *g* for 15 min with the brake on. Serum was then aliquoted into cryotubes and stored at −80°C until use. All the serum samples used in this study are from wave 1 of HANDLS and were collected, stored and processed using the same protocol.

### 2.2 Inflammatory protein measurements

Nine different biomarkers (IFN‐γ, IL‐6, TNF‐α trimer, E‐Selectin, MCP‐1, sRAGE, SAA, P‐Selectin, fibrinogen) were chosen based on their potential roles as inflammatory markers in age‐associated diseases and conditions. These nine inflammatory proteins were assayed from serum using a SP‐X Multiplex Planar Immunoassay on the Quanterix SP‐X platform (Billerica, MA, USA). Values outside the limit of detection (LOD) were removed; only IFN‐γ (2.6%) and TNF‐α trimer (15.9%) had more than 1% outside the LOD. Due to the higher number of values outside the LOD for TNF‐α, we removed this inflammatory protein from the mortality analysis. The inflammatory proteins had a mean intra‐assay coefficient of variation (CV) of 8.5% and inter‐assay CV of 8.0% (Table S1). Due to all the protein distributions being positively skewed, values were log_2_ transformed for statistical analysis. The high and low levels of inflammatory proteins used in the figures are the 25th and 75th percentiles of the log_2_ transformed values.

### 2.3 Mortality assessment

Mortality data were derived from the National Death Index from enrollment (2004–2009) through December 31, 2020. There were 236 deaths with an average follow‐up time of 12.7 years (median: 13.3 years, maximum: 16.4 years).

### 2.4 Statistics

Multivariable Cox proportional hazards regression models were used to examine how each inflammatory protein, along with race, sex and poverty status, were related to all‐cause mortality. Time was measured by the exact age at entrance and exit of the study, which was either date of death or censored at December 31, 2020. Using age as the measure of time, as opposed to time‐on‐study with age as a covariate, can avoid biased estimates^33^ for cohorts enrolled across a range of ages such as HANDLS. All inflammatory proteins were examined, starting with all possible interactions among each inflammatory protein with race, sex and poverty status, using backward selection to determine the most parsimonious model. Final models were confirmed with forward variable selection and examined for adequate model fit through examining Schoenfeld residuals. Hazard ratios (HR) are provided with 95% confidence intervals and a two‐sided *p* < .05 significance level was used for all analyses. Data management and analyses were performed in R software version 4.2.^34^ HRs for significant interactions were determined using the contrast function in the R package rms.

## 3 RESULTS

Overall, 1122 HANDLS participants were included in this study; 44% were White and 55% were African–American adults with a mean age of 47.7 and 47.9 years, respectively at baseline (Table 1). The cohort consisted of 51.4% women and 48.6% men. In this cohort, African–American participants were more likely to be living below the 125% poverty line compared to White participants. There was no significant difference in the overall number of deaths between White adults and African–American adults.

**TABLE 1:** Description of the study cohort.

	Overall (N = 1122)	White (N = 506)	African–American (N = 616)	*p*‐Value
**Age, mean (SD)**	47.8 (9.23)	47.7 (9.07)	47.9 (9.38)	.791
**Sex, n (%)**				.265
Women	577 (51.4%)	270 (53.4%)	307 (49.8%)	
Men	545 (48.6%)	236 (46.6%)	309 (50.2%)	
**Poverty Status, n (%)**				<.001
Above	700 (62.4%)	364 (71.9%)	336 (54.5%)	
Below	422 (37.6%)	142 (28.1%)	280 (45.5%)	
**Deaths, n (%)**	236 (21%)	98 (19%)	138 (22%)	.243
**E‐Selectin (ng/mL), mean (SD)**	81.72 (43.61)	76.66 (35.55)	85.88 (48.90)	<.001
**Fibrinogen (μg/mL), mean (SD)**	12.20 (93.56)	11.59 (111.96)	12.70 (75.01)	<.001
**IFN‐γ (pg/mL), mean (SD)**	1.05 (2.91)	1.25 (3.29)	0.881 (2.55)	<.001
**IL‐6 (pg/mL), mean(SD)**	3.40 (6.80)	3.26 (6.44)	3.50 (7.09)	.972
**MCP‐1 (pg/mL), mean (SD)**	963 (446)	1160 (408)	802 (410)	<.001
**P‐Selectin (ng/mL), mean (SD)**	493 (179)	516 (195)	474 (163)	.001
**SAA (μg/mL), mean (SD)**	37.51 (217.01)	20.93 (55.69)	51.18 (288.10)	.818
**sRAGE (pg/mL), mean (SD)**	667 (489)	843 (480)	522 (447)	<.001
**TNF‐α trimer (pg/mL), mean (SD)**	4.48 (13.0)	4.68 (12.9)	4.31 (13.1)	.014

*p*‐values are for tests between racial groups; categorical variable (sex, poverty status) tests are Chi‐squared tests of independence, and continuous variables tests are Student's *t*‐test. *p*‐values for inflammatory proteins were based on the log_2_ transformed variables (see Supporting Information Table S2).

Inflammatory protein levels overall and by race are listed in Table 1. Inflammatory protein levels were positively skewed and thus were log_2_ transformed for statistical analysis and values are listed in Table S2. Serum levels of E‐selectin and fibrinogen were significantly higher in African–American adults. IFN‐γ, TNF‐α trimer, MCP‐1, sRAGE and P‐selectin were significantly higher in White participants compared to African–American participants (Table 1). There were no significant differences in SAA or IL‐6 serum levels between White and African–American participants.

**FIGURE 1:**
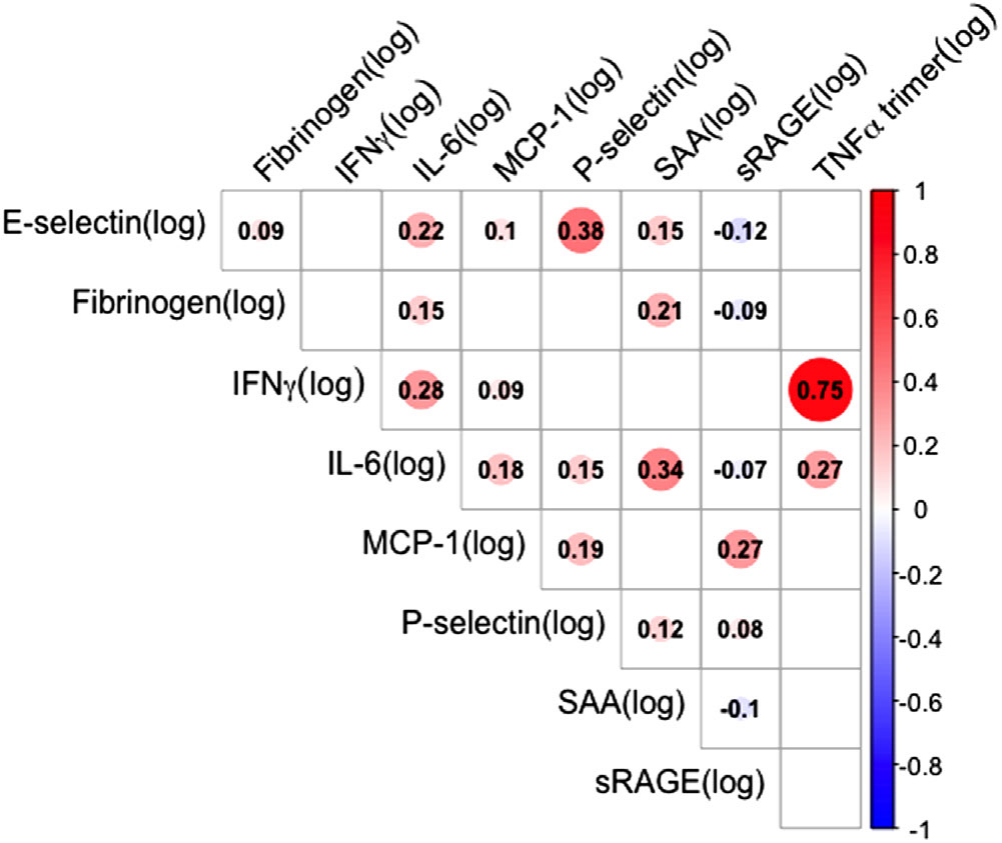
Correlations among inflammatory proteins. Pearson correlation coefficients are displayed for each inflammatory protein pair using the log_2_ transformed variables (original values in units of pg/mL). Only correlation coefficients that were significant at *p* < 0.05 are shown. Circle size indicates the strength of the correlation with red indicating a positive correlation, and blue a negative one.

Using Pearson correlation, we found that there were various significant relationships between the inflammatory markers (Figure 1). This analysis shows that the inflammatory markers that we chose to assay may reflect both similar and different inflammatory pathways.

**FIGURE 2:**
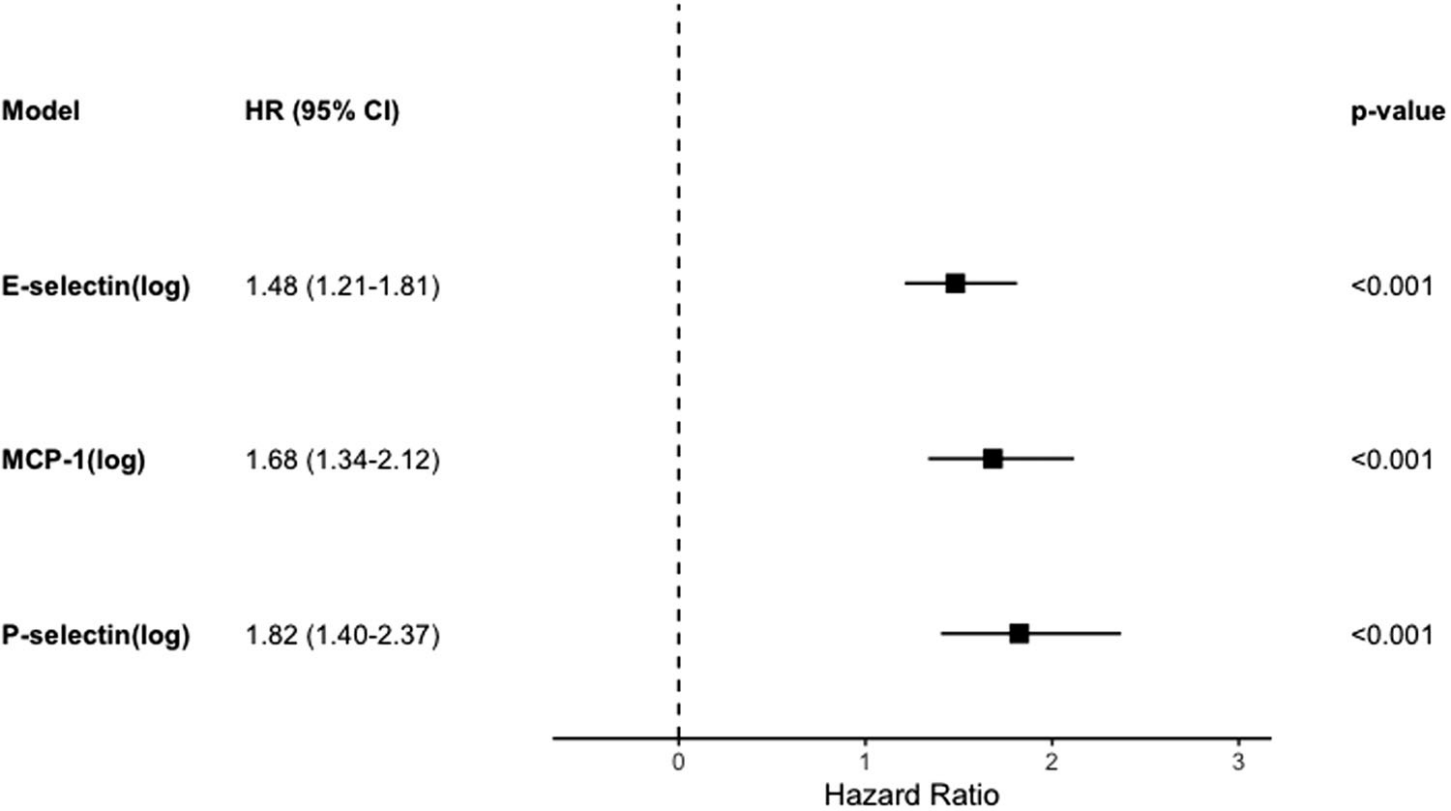
Hazard Ratios for separate Cox regression models for P‐selectin, E‐selectin and MCP‐1 on survival. Results of Cox Regressions for overall mortality, displaying the hazard ratio and 95% confidence interval (CI) for each coefficient. Each model (row) represents a separate regression model including the inflammatory protein listed as an independent variable, along with race, sex and poverty status. *p*‐values are for the listed inflammatory protein hazard ratio.

Inflammatory markers were assayed from samples at study baseline (2004–2009) and mortality was ascertained through the end of 2020, with an average follow‐up time of 12.7 years. We examined the mortality risk associated with sex, race, poverty status and each inflammatory protein, as well as possible interactions. There was a significant association between E‐selectin (HR: 1.48 [1.21–1.81] *p* < .001), MCP‐1 (HR: 1.68 [1.34–2.12] *p* < .001) and P‐selectin (HR: 1.82 [1.40–2.37] *p* < .001) and mortality (Figure 2). Higher levels of these inflammatory proteins were significantly associated with increased mortality risk. Below‐poverty status and male sex were also significantly associated with an increased risk of mortality in all three models (Supporting Information Table S3). Race was only significant in the model with MCP‐1, with African–American participants having a higher risk of mortality than White participants (Supporting Information Table S3).

**FIGURE 3:**
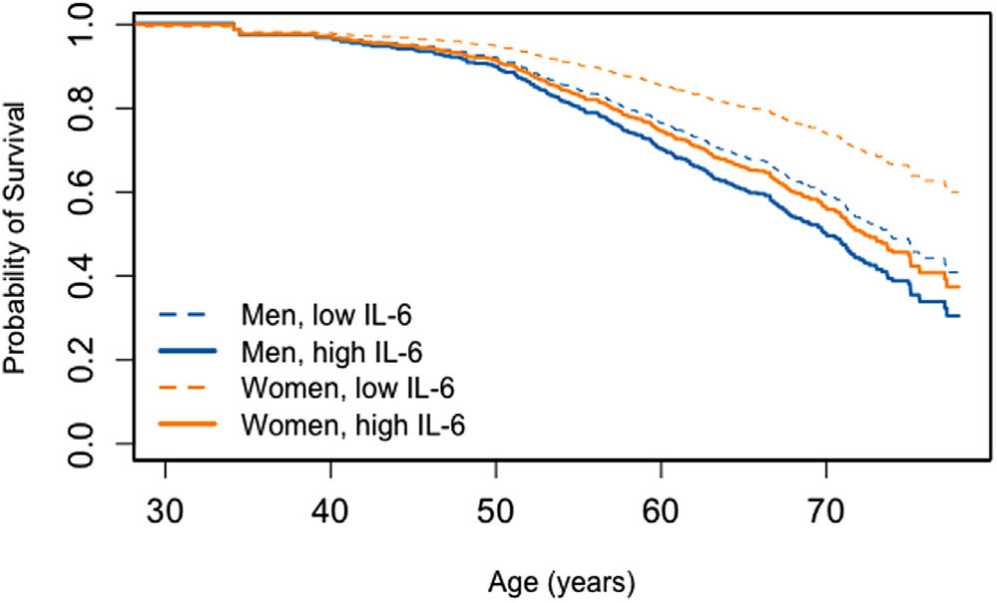
Survival probability by IL‐6 level and sex across age. Probability of survival from Cox Regressions for overall mortality, displaying the survival curve for the interaction of sex and IL‐6. The regression model also included poverty status and race (Supporting Information Table S4). Low and high IL‐6 values are the first and third quartile of the log_2_ transformed values respectfully.

**FIGURE 4:**
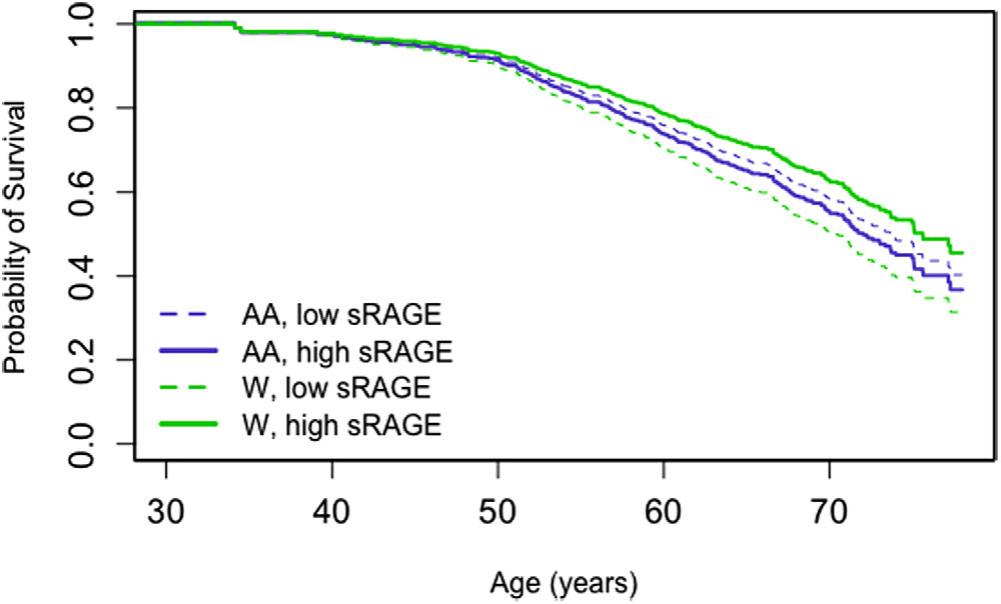
Survival probability by sRAGE level and race across age. Probability of survival from Cox Regressions for overall mortality, displaying the survival curve for the interaction of race (AA = African–American, W = White) and sRAGE. The regression model also included poverty status and sex (Supporting Information Table S4). Low and high sRAGE values are the first and third quartile of the log_2_ transformed values respectfully.

Two inflammatory proteins were associated with mortality through interactions with covariates. We found a significant interaction between sex and IL‐6 levels for overall mortality (Figure 3, *p* = .010). For both men and women, higher IL‐6 levels were associated with a high risk for mortality. Men with higher levels of IL‐6 had an increased risk of mortality compared with men with lower levels of IL‐6 (HR: 1.33 [1.11–1.59] *p* = .002). Women with higher levels of IL‐6 also had an increased risk of mortality compared to women with lower levels of IL‐6 (HR: 1.95 [1.55–2.45] *p* < .001), but this difference was significantly greater than that for men. In addition, we uncovered a significant interaction between race and sRAGE levels (Figure 4, *p* = .010). White participants with high levels of sRAGE had significantly lower risk of mortality than White participants with low levels of sRAGE (HR: .68 [.51–.90] *p* = .008), while African–American participants had similar risk of mortality across sRAGE levels (HR: 1.10 [.86–1.41] *p* = .431). Living below poverty status and male sex were also significantly associated with increased risk of mortality in these two models (Supporting Information Table S4).

## 4 DISCUSSION

Here, we examined the relationship of nine different inflammatory markers with risk for mortality in a middle‐aged, socioeconomically diverse cohort of African–American and White men and women. We found significant differences in inflammatory‐related protein serum levels between African–American and White middle‐aged adults. E‐selectin and fibrinogen were significantly higher in African–American adults. IFN‐γ, TNF‐α trimer, MCP‐1, sRAGE and P‐selectin were significantly higher in White participants compared to African–American participants. Higher levels of E‐selectin, MCP‐1 and P‐selectin were associated with increased mortality risk. Furthermore, there was a significant interaction between sex and IL‐6 with mortality. IL‐6 cytokine levels were associated with an increased risk of mortality, an association that was significantly greater in women than men. In addition, White participants with high levels of sRAGE had a significantly lower risk of mortality than White participants with low levels of sRAGE.

E‐selectin plays an important role in binding and recruiting of leukocytes to the activated endothelial layer, and higher soluble circulating levels may reflect endothelial inflammatory processes such as sites of atherosclerotic plaques. Our findings that E‐selectin was associated with mortality are consistent with other previous findings. For example, higher circulating E‐selectin levels predicted future death due to cardiovascular events in patients with coronary artery disease^24^ and in chronic heart failure patients with diabetes.^25^ However, E‐selectin was not associated with mortality in an older (mean age = 69 years) cohort of White patients with carotid atherosclerosis.^35^ In a recent study using data from the Social Environment and Biomarkers of Aging Study, the authors examined intrinsic capacity (IC), which combines domains of all physical and mental capacities and is thought to indicate the functional reserve of an individual in the aging process.^36^ This composite score was then compared to biological markers, and it was found that low IC (indicating low functional reserve) was associated with high IL‐6 and high E‐selectin, along with low serum albumin and low folate, and a low IC predicted a 4‐year mortality risk.^36^ Interestingly, E‐selectin was not associated with the IC score solely in the older population (>60 years).^36^ These data are consistent with our findings that E‐selectin was associated with mortality at midlife. E‐selectin serum levels were higher in African–Americans in our study. Previous studies have also found higher levels of E‐selectin in African–American adults. However, in the Atherosclerosis Risk in Communities Study only African–Americans with documented coronary heart disease and carotid atherosclerosis had elevated E‐Selectin levels.^37^ However, in a study of African ancestry and White adults there was no significant difference in E‐selectin levels.^38^ Recent research reveals that a common loss‐of‐function missense variant of fucosyltransferase 6 at chromosomal location Chr19p13 is associated with higher levels of soluble E‐selectin. This variant is more common in African ancestry populations, suggesting that differences may be related to the prevalence of the genotype in the population studied.^39^

Like E‐selectin, P‐selectin is involved in the binding of leukocytes to the activated endothelium. It is also expressed on activated platelets and plays a role in platelet recruitment and aggregation to sites of injury during inflammation. Circulating P‐selectin levels have been associated with vascular and thrombotic diseases.^40^ Here, we reported that P‐selectin was associated with mortality. This is consistent with another report showing that high P‐selectin levels were associated with a greater risk of all‐cause mortality in a cohort of patients with heart failure with preserved ejection fraction (mean age = 74 years).^26^ In patients with atrial fibrillation, P‐selectin was associated with mortality in older (mean age = 78 years) participants in the Rotterdam study^41^ but not in another study (median age = 69 years).^42^ P‐selectin was not associated with all‐cause mortality in the Framingham Heart Study (mean age = 61 years; n = 3035).^29^ However, this was an older, largely White adult cohort. This highlights the gap in our knowledge about the relationship between P‐selectin and mortality, especially in diverse, middle‐aged, community‐based cohorts since most studies have focused on White, older populations with vascular disease. Our finding that P‐selectin is higher in White adults dovetails with the data from the Wandsworth Heart and Stroke Study, which demonstrated significantly lower levels of P‐selectin in middle‐aged individuals of African ancestry compared to White individuals.^38^

MCP‐1/CCL2 is secreted by a variety of cell types and acts as a chemokine to attract monocytes. Importantly, our results showed that MCP‐1 was associated with mortality in this middle‐aged cohort. This is in agreement with a recent meta‐analysis of MCP‐1 levels in seven population‐based cohorts (n = 21 401), which reported that higher MCP‐1 was associated with long‐term cardiovascular mortality.^22^ This is an important study providing a link between MCP‐1 and cardiovascular mortality, likely through promoting atherosclerosis, in populations without overt cardiovascular disease. It should be noted that most participants in this study were White adults, although Black (7.6%) and Hispanic (4.2%) individuals were included, and race and ethnicity was included as a covariate in the analysis. In the multi‐ethnic Dallas Heart Study, MCP‐1 was independently associated with all‐cause mortality in the context of chronic kidney disease.^43^ We found that MCP‐1 levels were higher in White adults compared to African–American adults. This replicates findings from the Dallas Heart study that reported lower MCP‐1 levels in African–American study participants.^44^ MCP‐1 levels are influenced by the presence of a single nucleotide polymorphism in the 5,10‐methylenetetrahydrifolate reductase gene.^45^ MCP‐1 has also been proposed as a biomarker of biological aging in mouse models and frail older adults with aortic stenosis.^23^ Previously, we reported that levels of extracellular vesicle‐associated MCP‐1 were higher with mortality in a diverse cohort from the HANDLS study.^46^ In this study, we did not find any differences in EV‐associated levels of MCP‐1 with race, sex, or poverty. Therefore, this work builds upon these studies to implicate MCP‐1 as a marker of mortality in a diverse cohort.

Over 20 years ago, it was reported that IL‐6 levels were associated with mortality in White elderly adults (mean age = 77.8 years),^47^ and in older, disabled women (mean age = 77.8 years).^16^ Since these initial findings, IL‐6 has been reported to be associated with mortality in older cohorts (rev in ^48^) and in middle‐aged to older predominantly White adults in the Framingham Heart Study (mean age: 61 ± 9 years).^29^ Very few studies have examined whether there were sex differences. Harris et al., stratified by sex and found that women, but not men, had an increased mortality risk at high levels of IL‐6.^47^ In the Framingham Heart Study and other studies, there were no reported differences with sex, or sex was not examined.^29^, ^48^ Consistent with our data that men with high IL‐6 had the lowest survival probability, IL‐6 was associated with all‐cause mortality only in elderly men but not women (age: 65−83 years) in the MEMO study.^49^ In a study of elderly men (mean age = 77.7 years), IL‐6 was associated with all‐cause and cardiovascular mortality.^50^ Therefore, few studies have reported in population‐based studies whether there are sex‐specific associations between IL‐6 and mortality, especially in studies that contain both sexes. However, it is interesting to speculate that sex differences in IL‐6 levels may explain the differential mortality risk in men versus women. Serum IL‐6 levels were not significantly different between African–American and White adults in our study. This differs from some previously published investigations, which found that serum IL‐6 levels were higher in African–American compared to White adults.^45^, ^51^ However, there is one study that found lower levels of serum IL‐6 in Black adults.^52^ These differences may be related to varying frequencies for the G/G genotype within the IL‐6 gene, which is associated with higher protein expression.^53^

In our study, we found that White participants with high levels of sRAGE had a significantly lower risk of mortality than White participants with low levels of sRAGE, while African–American participants had similar risk of mortality across sRAGE levels. RAGE is a pattern‐recognition receptor that exists as a membrane‐bound form but also as a soluble form (sRAGE), which is generated through either proteolytic cleavage between the extracellular and transmembrane regions or through alternative splicing. sRAGE acts as a decoy receptor by binding to advanced glycation end‐products (AGEs), damage‐associated molecular pattern molecules and other ligands for RAGE. Thus, it is thought that sRAGE plays a role in counteracting inflammation and reducing oxidative stress. An alternative hypothesis is that sRAGE levels may reflect chronic RAGE stimulation and heightened inflammation. Data from human populations reveal a complex association of sRAGE levels with age‐associated diseases and mortality.^27^ In general, levels of sRAGE are higher with diabetes mellitus^54^, ^55^ and renal disease^56^ but lower in cardiometabolic conditions.^54^, ^55^ Various results have been reported regarding sRAGE and mortality in human populations,^27^ and in general, were higher with mortality in cohorts with chronic disease states or cause‐specific deaths.^27^ Consistent with other reports, we found lower levels of sRAGE in African–American adults compared to White adults.^44^, ^57^, ^58^ However, in these studies, whether differences in sRAGE were associated with mortality in the context of race were not assessed. Therefore, our data builds upon the current findings examining sRAGE and mortality and identifies race‐specific differences in the relationship between sRAGE and mortality.

SAA is an acute‐phase response protein produced largely by the liver in response to events such as inflammation, infections and trauma.^59^ In our study, we did not find any associations between SAA and mortality. This is in contrast to findings from the Ludwigshafen Risk and Cardiovascular Health (LURIC) study, where in patients at risk for cardiovascular disease, higher SAA was associated with all‐cause and cardiovascular mortality.^28^ SAA can be incorporated into high‐density lipoproteins interfering with its function and in this study, SAA abrogated the protective effects of HDL on mortality.^28^ Therefore, the vasoprotective and biological effects of HDL may depend on SAA levels. In patients with subclinical carotid atherosclerosis, SAA levels were associated with mortality but not when these findings were adjusted for another acute phase reactant protein, high sensitivity C‐reactive protein (hsCRP).^60^ These data are consistent with the earlier work by Ridker and colleagues that reported that hsCRP was a better predictor of CVD events (including death) than SAA.^61^ However, in women referred for coronary angiography for evaluation of suspected myocardial ischemia as part of the Women's Ischemia Syndrome Evaluation (WISE) study, SAA was an independent predictor of cardiovascular events (including death) (mean age: 58 years; 18% non‐White).^62^ SAA is also associated with cardiovascular risk factors including obesity,^63^ type 2 diabetes,^64^ and renal disease.^65^ For example, SAA was associated with mortality in hemodialysis patients^65^ and it was associated with cardiac events but not mortality in individuals with type 2 diabetes on hemodialysis.^66^ SAA levels were associated with end‐stage renal disease but not mortality in American Indians with type 2 diabetes.^67^ In this population, higher SAA was associated with a reduced risk of ESRD,^67^ suggesting the need for interrogating inflammatory markers in diverse populations. In summary, although SAA has been examined mostly in the context of risk factors for CVD and mortality, data are spurious on the association of SAA to mortality in community‐based cohorts, with the exception of the LURIC study. This may be reflective in our lack of association of SAA with mortality in our cohort.

There were additional race‐related differences in inflammatory biomarkers between African–American and White adults. We have replicated the finding that African–American adults have higher levels of fibrinogen than White adults. Several previous studies found that thrombotic biomarker profiles differ between African–American and White adults, with African–American adults having higher levels of prothrombotic factors including fibrinogen.^68^, ^69^ Although fibrinogen, as a marker of thrombosis and inflammation, has been associated with overall all‐cause mortality and in cardiovascular risk‐related mortality, it was not a significant risk factor for mortality in this middle‐aged cohort.^29^, ^30^, ^31^ This discrepancy may lie in the fact that most of these other cohorts were examined in relation to cardiovascular‐related disease or events. IFN‐γ was higher among our White study participants, consistent with findings in another study.^52^ In contrast, other investigators found that African–American women had significantly higher mean levels of IFN‐γ then White women.^70^ These findings may be related to the prevalence of the IFN‐γ A/A genotype, which is associated with low gene expression.^53^ This genotype is more common in African–American individuals. Resolution of these conflicting findings will require assessments done in the context of cohort genotypes. TNF‐α levels were lower in African–American compared to White adults in our study, in agreement with another study.^52^ However, three studies found no difference in TNF‐α levels between African–American and White adults.^44^, ^71^, ^72^

By selecting inflammatory markers for our panel from a variety of inflammatory pathways, our work highlights the fact that many etiologies of inflammation play a role in mortality risk. In addition, we are providing additional evidence that some of the proteins associated with the SASP are also associated with poor health outcomes, in this case, mortality. SASP, comprised of factors that are significantly altered between pre‐senescent and senescent cells includes interleukins (e.g., IL‐6), chemokines (MCP‐1) and other inflammatory molecules (IFN‐γ), soluble receptors (TNF‐α), non‐protein soluble protein factors and insoluble factors,^13^ has been associated with chronic disease, advanced biologic age, frailty, medical risk and other adverse health outcomes.^21^ We have demonstrated that even at middle age, senescence‐associated proteins are linked with mortality, supporting the hypothesis that health disparities related to mortality may be propelled in part by the accelerated aging phenotype as well as inflammation.

This study has several strengths and limitations. It is a large‐scale study of inflammatory markers and mortality over more than twelve years in a middle‐aged community‐based socioeconomically diverse cohort of African–American and White adults. Most biomarker and mortality studies utilize older, predominantly White cohorts or cohorts that are focused on a specific disease. Our approach allowed us to examine the association of inflammatory proteins with mortality, race, sex and poverty earlier in the life span. Our analysis did not account for other health‐related baseline attributes (i.e., obesity, diabetes, ASCVD), which may influence inflammation. Our study has other limitations as well. Here, we used a targeted, hypothesis‐driven approach in choosing the nine inflammatory markers to profile. However, this approach is restrictive versus other unbiased proteomic approaches. Our findings are also limited by the use of baseline demographic information and inflammatory marker measurements at a single time without serial measurements of inflammatory markers. Therefore, variations in the stability of these markers may limit their predictive power regarding mortality. Nevertheless, we report significant associations of five proteins with mortality. Given the limited number of deaths in the cohort, we were unable to perform cause‐specific mortality associations with the different markers. We were also unable to balance the cohort across poverty status. In addition, our data should be interpreted with caution, as it is not known whether these markers play a causative role in mortality or merely are altered by compensatory mechanisms responding to pathway activation.

In this prospective study, we found that higher levels of E‐selectin, MCP‐1 and P‐selectin were associated with mortality in a middle‐aged cohort of African–American and White men and women. We also found that high levels of IL‐6 were associated with a greater risk of mortality, especially in women. The association of sRAGE with mortality differed in African–American and White adults. These data indicate that inflammatory processes are associated with mortality at midlife and suggest that interventions targeting these pathways may be beneficial in at‐risk middle‐aged populations. However, additional studies are warranted to provide a direct link between these inflammatory proteins and specific interventions.

## FUNDING INFORMATION

This work was supported by the National Institute on Aging Intramural Research Program, National Institutes of Health, Project AG 000513.

## CONFLICT OF INTEREST STATEMENT

The authors declare that they have no competing interests.

## DATA AVAILABILITY STATMENT

The datasets generated and analyzed during the current study are available from the corresponding author on reasonable request through the HANDLS website https://handls.nih.gov/.

## Supporting information

Supporting Information
