# Supplementary material for: Inflammatory proteins are associated with mortality in a middle‐aged diverse cohort
Source: Clin Transl Med. 2023 Sep 24;13(9):e1412. doi: 10.1002/ctm2.1412 (PMC10518496; doi:10.1002/ctm2.1412)
Supplement: Supplementary file 1 — Supporting Information [file CTM2-13-e1412-s001.docx]

**Supporting Information**

**Noren Hooten et al., Inflammatory proteins are associated with mortality in a middle-aged diverse cohort**

**Table S1. Inflammatory protein inter- and intra-assay coefficients of variation**

| **Inflammatory Protein** | **Inter-assay CV (%)** | **Intra-assay CV (%)** |
| --- | --- | --- |
| **E-Selectin** | **2.2** | **15.8** |
| **Fibrinogen** | **3.4** | **13.7** |
| **IFNγ** | **8.4** | **3.8** |
| **IL-6** | **6.7** | **4.8** |
| **MCP-1** | **7.3** | **5.5** |
| **P-Selectin** | **16.5** | **6.4** |
| **SAA** | **9.3** | **9.2** |
| **sRAGE** | **6.4** | **11.7** |
| **TNF-α trimer** | **11.6** | **6.0** |

Mean coefficient of variation (CV) for precision for this cohort

**Table S2. Log_2_ transformed inflammatory protein values by race**

|  | **Overall (N=1122)** | **White (N=506)** | **AfrAm (N=616)** | **P-value** |
| --- | --- | --- | --- | --- |
| **E Selectin (log), mean(sd)** | 16.2 (0.654) | 16.1 (0.644) | 16.2 (0.655) | <0.001 |
| **Fibrinogen (log), mean(sd)** | 22.5 (1.15) | 22.3 (1.07) | 22.6 (1.18) | <0.001 |
| **IFNγ (log), mean(sd)** | -2.05 (2.40) | -1.76 (2.44) | -2.30 (2.34) | <0.001 |
| **IL-6 (log), mean(sd)** | 1.16 (1.13) | 1.16 (1.09) | 1.16 (1.16) | 0.972 |
| **MCP-1 (log), mean(sd)** | 9.78 (0.610) | 10.1 (0.473) | 9.52 (0.584) | <0.001 |
| **P Selectin (log), mean(sd)** | 18.8 (0.524) | 18.9 (0.544) | 18.8 (0.501) | 0.001 |
| **SAA (log), mean(sd)** | 22.9 (2.02) | 22.9 (1.74) | 22.9 (2.23) | 0.818 |
| **sRAGE (log), mean(sd)** | 9.04 (1.04) | 9.48 (0.892) | 8.67 (1.01) | <0.001 |
| **TNFα trimer (log), mean(sd)** | -0.653 (2.85) | -0.411 (2.77) | -0.869 (2.91) | 0.014 |
|  |  |  |  |  |

**Table S3. Hazard Ratios for overall mortality and 95% CI for Cox Regression models for six inflammatory proteins**

|  | **E Selectin**  **(n= 1121)** | **Fibrinogen^1^**  **(n=1115)** | **IFNγ**  **(n=1093)** | **MCP-1**  **(n=1121)** | **P Selectin**  **(n= 1120)** | **SAA**  **(n=1120)** |
| --- | --- | --- | --- | --- | --- | --- |
| Poverty Status (below) | **1.66**  **(1.28, 2.16)** | **1.74**  **(1.34, 2.26)** | **1.83**  **(1.40, 2.38)** | **1.74**  **(1.34, 2.25)** | **1.83**  **(1.41, 2.37)** | **1.76**  **(1.36, 2.29)** |
| Sex (male) | **1.30**  **(1.00, 1.68)** | 1.03  (0.69, 1.53) | **1.33**  **(1.02,1.72)** | **1.34**  **(1.04, 1.74)** | **1.30**  **(1.00, 1.69)** | **1.39**  **(1.07, 1.80)** |
| Race (African American) | 0.99  (0.76, 1.29) | 0.75  (0.51, 1.11) | 1.04  (0.80, 1.37) | **1.38**  **(1.03, 1.85)** | 1.10  (0.84, 1.43) | 1.04  (0.80, 1.35) |
| Inflammatory Protein | **1.48**  **(1.21, 1.81)** | 1.06  (0.96, 1.18) | 1.00  (0.94, 1.05) | **1.68**  **(1.34, 2.12)** | **1.82**  **(1.40, 2.37)** | 1.06  (1.00, 1.13) |

^1^ This model also included a significant interaction for race and sex (HR: 1.72 (1.01-2.92)).

Results of separate Cox Regressions for overall mortality, displaying the hazard ratio and 95% confidence interval (CI) for each coefficient. Each column represents a separate regression model including the inflammatory protein listed as an independent variable. The outcome is overall mortality. Sample size for each model is presented in the column heading (n). Hazard ratios are in bold when significant at p<0.05.

**Table S4. Hazard Ratios for overall mortality and 95% CI for Cox Regression models for IL-6 and sRAGE**

|  | **IL-6**  **(n=1122)** | **RAGE**  **(n=1120)** |
| --- | --- | --- |
| Poverty Status (below) | **1.75 (1.35, 2.27)** | **1.76 (1.36, 2.29)** |
| Sex (male) | **2.02 (1.31, 3.09)** | **1.37 (1.06, 1.77)** |
| Race (African American) | 1.09 (0.84, 1.42) | **0.04 (0, 0.46)** |
| Inflammatory Protein | **1.65 (1.39,1.97)** | **0.76 (0.62, 0.93)** |
| Inflammatory Protein *Sex | **0.75 (0.60, 0.93)** | - |
| Inflammatory Protein *Race | - | **1.42 (1.09, 1.85)** |

Results of Cox Regressions for overall mortality, displaying the hazard ratio and 95% confidence interval (CI) for each coefficient. Each column represents a separate regression model including the cytokine listed as an independent variable. Sample size for each model is presented in the column heading (n). Hazard ratios are in bold when significant at p<0.05.
